# Aberrant expression and DNA methylation of lipid metabolism genes in PCOS: a new insight into its pathogenesis

**DOI:** 10.1186/s13148-018-0442-y

**Published:** 2018-01-12

**Authors:** Jie-Xue Pan, Ya-Jing Tan, Fang-Fang Wang, Ning-Ning Hou, Yu-Qian Xiang, Jun-Yu Zhang, Ye Liu, Fan Qu, Qing Meng, Jian Xu, Jian-Zhong Sheng, He-Feng Huang

**Affiliations:** 1 The International Peace Maternity and Child Health Hospital, School of Medicine, Shanghai Jiao Tong University, Shanghai, 200030 China; 2 The Key Laboratory of Reproductive Genetics, Ministry of Education (Zhejiang University), Hangzhou, Zhejiang 310006 China; 3 Department of Pathology and Pathophysiology, School of Medicine, Zhejiang University, Hangzhou, 310058 China; 4 Reproductive Medicine Center, The First Affiliated Hospital of Wenzhou Medical University, Wenzhou, 325000 China

**Keywords:** Polycystic ovary syndrome, RNA-seq, Methylation, Hyperandrogenism, Synthesis of lipid and steroid

## Abstract

**Background:**

Polycystic ovary syndrome (PCOS), whose etiology remains uncertain, is a highly heterogenous and genetically complex endocrine disorder. The aim of this study was to identify differentially expressed genes (DEGs) in granulosa cells (GCs) from PCOS patients and make epigenetic insights into the pathogenesis of PCOS.

**Results:**

Included in this study were 110 women with PCOS and 119 women with normal ovulatory cycles undergoing in vitro fertilization acting as the control group. RNA-seq identified 92 DEGs unique to PCOS GCs in comparison with the control group. Bioinformatic analysis indicated that synthesis of lipids and steroids was activated in PCOS GCs. 5-Methylcytosine analysis demonstrated that there was an approximate 25% reduction in global DNA methylation of GCs in PCOS women (4.44 ± 0.65%) compared with the controls (6.07 ± 0.72%; *P* < 0.05). Using MassArray EpiTYPER quantitative DNA methylation analysis, we also found hypomethylation of several gene promoters related to lipid and steroid synthesis, which might result in the aberrant expression of these genes.

**Conclusions:**

Our results suggest that hypomethylated genes related to the synthesis of lipid and steroid may dysregulate expression of these genes and promote synthesis of steroid hormones including androgen, which could partially explain mechanisms of hyperandrogenism in PCOS.

**Electronic supplementary material:**

The online version of this article (10.1186/s13148-018-0442-y) contains supplementary material, which is available to authorized users.

## Background

Polycystic ovary syndrome (PCOS), characterized by chronic anovulation and hyperandrogenism, is one of the most prevalent endocrine disorders in women of reproductive age [1]. It is also thought to be the leading cause of anovulatory infertility [1]. There is no single etiologic factor that fully accounts for the pathogenesis of PCOS, and several lines of evidence demonstrate that PCOS is a complex and multifactorial disorder with a high degree of heritability [2]. The high degree of familial aggregation of PCOS suggests that genetic factor plays an important role in its etiology [3, 4]. Recent genetic studies have been performed without adding significant new knowledge to the field [5, 6].

There is evidence indicating that epigenetic alterations, including aberrant DNA methylation might contribute to the development of PCOS [7]. DNA methylation is a highly tissue-specific phenomenon that varies with time on the basis of environmental fluctuations [8]. It is involved in the stability of gene expression during organismal development [9, 10]. Specific genes such as *LHR* [11], *EPHX1* [12], and *CYP19A1* [13] have been demonstrated to be associated with PCOS with aberrant DNA methylation in distinct tissues. In a recent genome-wide study, Wang et al. reported that DNA methylation and gene expression differences exist between PCOS and non-PCOS ovaries [14]. Our previous work has also indicated that epigenetic alterations of several important transcription factors are involved in the follicular development in granulosa cells (GCs) that contribute to PCOS [7].

Ovarian androgen excess is the most typical pathological manifestation of PCOS [1, 15]. The mechanisms underlying androgen excess and abnormal follicular development in PCOS remain to be elucidated. In this study, we identified differentially expressed genes (DEGs) with global transcriptome sequencing and highlighted the abnormal biological pathways among these DEGs in the GCs of PCOS women. We compared global DNA methylation of GCs between control and PCOS groups. Furthermore, DNA methylation levels of selected genes were evaluated with MassArray EpiTYPER. The correlations between gene expression and differential methylation of CpG sites were also analyzed. The altered expression level of genes related to lipid metabolism and steroid synthesis combined with the disarranged DNA methylation in GCs of PCOS women suggest that abnormal lipid metabolism and steroid synthesis and epigenetic dysregulation play important roles in the pathogenesis of PCOS.

## Results

### Patient demographic data and clinical features

The clinical characteristics of PCOS women and controls are shown in Table 1. Enrolled cases and controls were of comparable age and fasting glucose levels. Significant differences between the two groups (*P* < 0.05) were found in menstrual cycle length, body mass index (BMI), antral follicle count (AFC), fasting insulin, HOMA-IR (homeostasis model assessment of insulin resistance), ratio of serum luteinizing hormone (LH)/follicle-stimulating hormone (FSH), serum TT (total testosterone) levels on the third day of spontaneous menstrual cycle, and concentration of TT, sex hormone-binding globulin (SHBG), FAI (free androgen index), and insulin in the follicular fluids (FFs). All these values mentioned above were greater for PCOS group than those in controls except for SHBG, which is lower in PCOS women.

**Table 1:** Demographic data and clinic characteristics of the IVF patients who participated in the study

Items	Control (*n* = 119)	PCOS (*n* = 110)	*P* value
Age	29.85 ± 0.37	29.11 ± 0.35	0.149
Body mass index	21.74 ± 0.29	22.79 ± 0.36	0.024
Cycle length (days)	29.00 ± 0.22	67.73 ± 6.40	< 0.001
Duration of infertility	4.24 ± 0.30	3.91 ± 0.26	0.416
Fasting glucose (mmol/L)	4.65 ± 0.04	4.75 ± 0.05	0.068
Fasting insulin (pmol/L)*	70.29 ± 2.99	100.19 ± 5.35	< 0.001
HOMA-IR*	2.00 ± 0.09	2.96 ± 0.16	< 0.001
Day 3 LH/FSH	0.72 ± 0.04	1.48 ± 0.09	< 0.001
Day 3 TT (nmol/L)	0.78 ± 0.03	3.02 ± 0.14	< 0.001
Day 3 DHEA-S (μmol/L)	6.61 ± 0.24	7.62 ± 0.33	0.013
Day 3 E_2_ (pmol/L)	130.69 ± 4.63	148.47 ± 7.96	0.055
AFC	9.92 ± 0.30	20.45 ± 0.39	< 0.001
Androstenedione (ng/ml) in FF^#^	6.62 ± 1.06	7.25 ± 1.28	0.345
TT (ng/ml) in FF^#^	4.87 ± 0.49	9.25 ± 1.21	0.022
SHBG (nmol/L) in FF^#^	57.70 ± 3.01	53.06 ± 3.14	0.042
FAI in FF^#^	31.63 ± 4.34	69.28 ± 13.81	0.024
Insulin (mU/L) in FF^#^	37.51 ± 3.13	48.45 ± 3.50	0.024
Retrieved oocyte number	12.58 ± 0.64	17.63 ± 0.89	< 0.001

Values are presented as mean ± SE. *P* value was determined by Student’s *t* tests

*HOMA-IR* homeostasis model assessment of insulin resistance, *Day 3* the third day of spontaneous menstrual cycle, *LH* luteinizing hormone, *FSH* follicle stimulating hormone, *TT* total testosterone, *DHEA-S* dehydroepiandrosterone-sulfate, *E*_*2*_ estradiol, *AFC* antral follicle count, *FF* follicular fluid, *SHBG* sex hormone binding globulin, *FAI* free androgen index

*n = 53 in control and *n* = 50 in PCOS

#*n* = 50 in control and *n* = 71 in PCOS

### Identification of DEGs in GCs between PCOS patients and controls

**Fig. 1:**
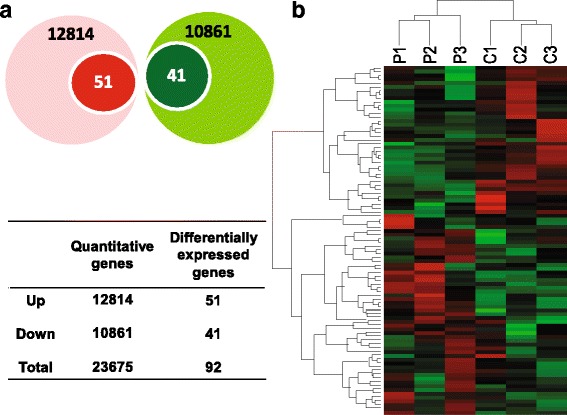
Differentially expressed genes in GCs between PCOS and control women. **a** Venn diagram showing the number of differentially expressed genes in GCs between the PCOS and control group. Red indicates upregulated genes, and green denotes downregulated genes. **b** Hierarchal clustering presentation of differentially expressed genes identified in GCs. Each row represents a single gene, and each column represents an experimental sample. Increasing green intensities denote genes that decrease in expression, and increasing red intensities denote gene that increases in expression in PCOS samples compared with control samples

To determine DEGs, GCs from three non-obese PCOS and three comparable control women were studied. Clinical characteristics of each participant are presented in Additional file 1: Table S3. According to the inspected RNA-seq data, all the quality control parameters were within the acceptable ranges. A total of 23,675 annotated Ensembl genes were detected and included in subsequent analysis (Additional file 2: Dataset S1), of which 12,814 genes were upregulated and 10,861 genes were downregulated in GCs of PCOS women (Fig. 1a). After applying statistical analysis (*P* < 0.05), a fold change (FC) criterion (fold change in gene expression levels between pathological and control GCs) > 1.5, and the false discovery rate (FDR) < 0.05, 92 genes were significantly differentially expressed in GCs of PCOS compared with controls, including 51 upregulated genes and 41 downregulated genes (Additional file 3: Dataset S2). Hierarchical clustering was performed on the dataset of DEGs (Fig. 1b). The results showed a striking separation between the two groups into two major opposite branches, indicating that the genes expressed in GCs of PCOS women were significantly different from those of controls.

### Bioinformatic analysis of DEGs

**Fig. 2:**
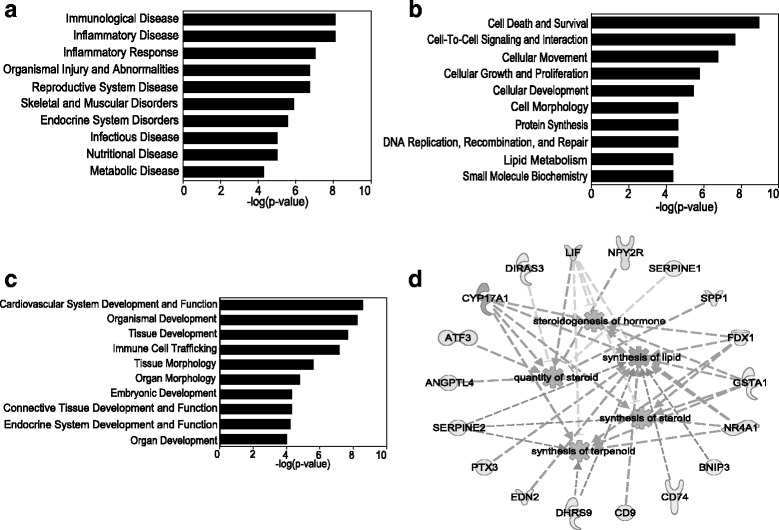
Bioinformatic analysis of the RNA-seq results. Ingenuity Pathway Analysis was used, which analyzes the expression data in the context of known biological response and regulatory networks as well as other higher order response pathways, to assign functional information and biological relevance. **a**–**c** Functional analysis of 92 DEGs. The top 10 influenced biofunctions are grouped by disease and disorder (**a**), molecular and cell functions (**b**), and physiological system development and functions (**c**). Results are shown as the negative logarithm of significance, which is a statistical score and a measure of the likelihood of the genes in a given network being found together as a result of chance, as determined by Fisher’s exact test. Downstream effects analysis of DEGs predicts activated lipid metabolism in GCs from PCOS women (**d**)

To better understand the potential function of the 92 DEGs, Ingenuity Pathway Analysis (IPA) was used to further analyze the expression data in the context of known biological responses as well as other higher order response pathways to assign functional information and biological relevance to these DEGs. These results are presented as negative logarithm of the significance level, which is a statistical score measuring the likelihood of the genes in a given network being found together through Fisher’s exact test. Based on the expression data of 92 DEGs, functional analysis showed that these DEGs were enriched in the category of diseases and disorder, molecular and cellular functions, and physiological system development and function (Fig. 2a–c). Within the category of diseases and disorders, the inflammatory response, reproductive system disease, and endocrine system disorders were included on the top ten related dysfunctions. Within the category of molecular and cell functions, cell death and survival, cell-to-cell signaling and interaction, cellular movement, and lipid metabolism were listed in the top related functions. Within the category of development and functions, the endocrine system development and function was also involved. The further downstream effect analysis of DEGs between the PCOS and control groups predicated that the synthesis of lipid was activated (*z* score = 2.014) in the PCOS group (Fig. 2d). The other lipid metabolism biological processes, such as steroidogenesis of hormone (*z* score = 1.253), the quantity of steroid (*z* score = 1.411), synthesis of steroid (*z* score = 1.253), and synthesis of terpenoid (*z* score = 1.253) might also be activated in the PCOS group (Fig. 2d). These alterations were in accordance with the hyperandrogenism of PCOS.

### Validation of DEGs related to lipid and steroid metabolism with qPCR

**Fig. 3:**
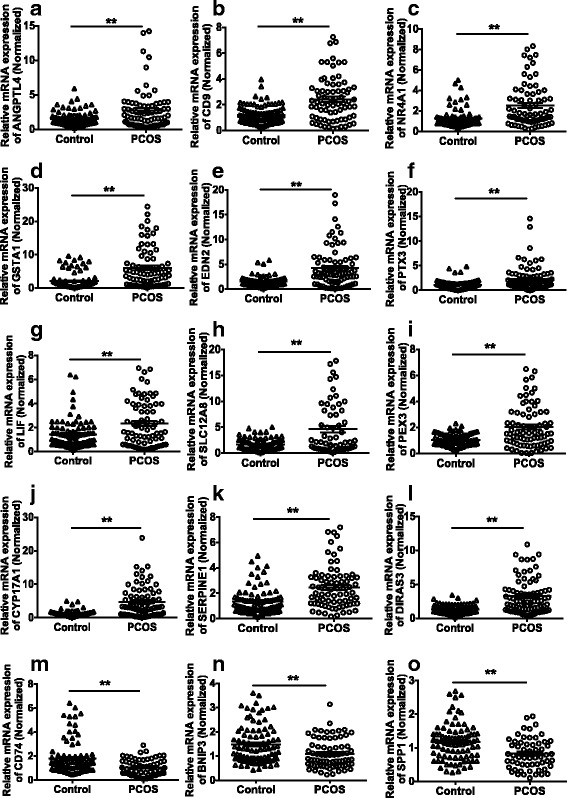
Verification of 15 differentially expressed genes related to lipid metabolism and steroid synthesis identified by RNA-seq. The mRNA expression levels for 15 DEGs in GCs of control (*n* = 93) and PCOS women (*n* = 79). **a**–**l** are upregulated and **m**–**o** are downregulated in transcription analysis and qPCR. Data are presented as mean ± SE. *P* value is determined by independent samples *t* test. *, **, *P* < 0.05 and *P* < 0.01, compared with the corresponding controls, respectively

To confirm the RNA-seq results, 12 upregulated genes and three downregulated genes related to lipid and steroid metabolism were identified with qPCR (Fig. 3). GCs from 93 control and 79 PCOS women were enrolled in this part of the study. The results of qPCR showed that the expression of related genes were in accordance with the RNA-seq analysis results.

### Global DNA methylation of GCs from PCOS women and controls

**Fig. 4:**
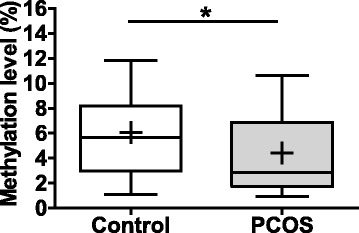
Global DNA methylation box plots. For each group, the bottom and top bars indicate the 10th and 90th percentiles, respectively. The bottom and top of each box indicate the 25th and 75th percentiles, the line through the middle of each box represents the median, and the mean value for each group is noted by (+) in each box. Thirty GC genomic DNA from controls and 39 GC genomic DNA from PCOS women were analyzed. *P* value is determined by Mann-Whitney *U* test. *, *P* < 0.05, compared with the controls

To compare the global DNA methylation level of the GCs between PCOS and controls, we assessed the levels of 5-mC in the genomic DNA of GCs by ELISA with antibody specific to 5-mC. As shown in Fig. 4, the global DNA methylation levels were 6.07 ± 0.72% and 4.44 ± 0.65% in the GCs from control and PCOS groups, respectively. We observed a significant reduction of approximately 25% in the level of 5-mC in GCs from PCOS women compared with controls (*P* < 0.05).

### MassArray EpiTYPER quantitative DNA methylation analysis of target genes

**Fig. 5:**
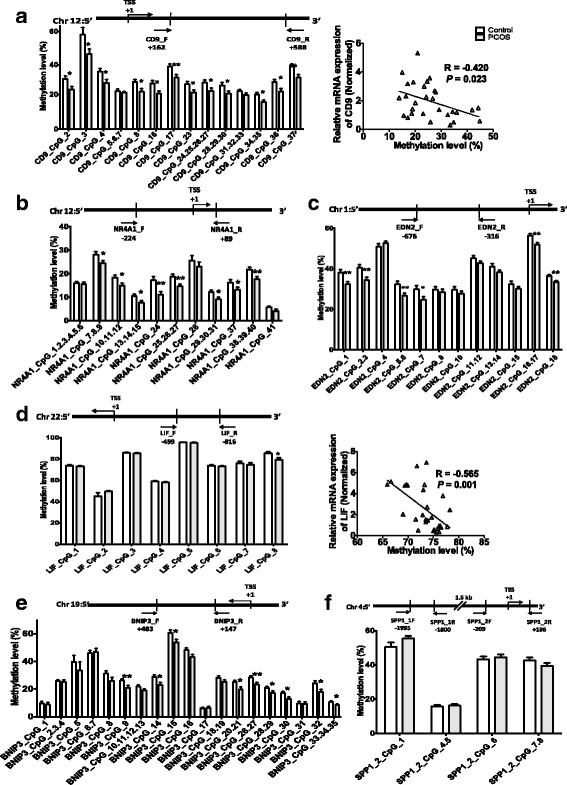
MassARRAY quantitative methylation analysis of promoter CpG sites of target genes. **a** The schematic diagram of amplicon in the promoter region of CD9 (up). The mean methylation levels for each CpG site of the *CD9* promoter (down). The correlations between *CD9* mRNA expression level and the average CpG site methylation level in the promoter of *CD9* (right). **b** The schematic diagram of amplicon in the promoter region of *NR4A1* (up). The mean methylation levels for each CpG site of the *NR4A1* promoter (down). **c** The schematic diagram of amplicon in the promoter region of *EDN2* (up). The mean methylation levels for each CpG site of the *EDN2* promoter (down). **d** The schematic diagram of amplicon in the promoter region of *LIF* (up). The mean methylation levels for each CpG site of the *LIF* promoter (down). The associations between average methylation level of *LIF* and its expression (right). **e** The schematic diagram of amplicon in the promoter region of *BNIP3* (up). The mean methylation levels for each CpG site of the *BNIP3* promoter (down). **f** The schematic diagram of amplicon in the promoter region of SPP1 (up). The mean methylation levels for each CpG site of the *SPP1* promoter amplified by primer 2 (down). Binding sties for each of the forward and reverse primers are shown as arrows below the diagram. Values are mean ± SE. Thirty-eight GC genomic DNA from controls and 47 GC genomic DNA from PCOS women were analyzed. *P* value is determined by Mann-Whitney *U* test. * represents *P* < 0.05, ** represents *P* < 0.01

We analyzed DNA methylation of 15 DEGs related to lipid metabolism and synthesis of steroid hormones, including *GSTA1*, *CD74*, *ANGPTL4*, *CD9*, *NR4A1*, *EDN2*, *PTX3*, *LIF*, *SLC12A8*, *PEX3*, *CYP17A1*, *SERPINE1*, *DIRAS3*, *BNIP3*, and *SPP1*. With the UCSC Genome Browser (http://www.genome.ucsc.edu/) and online primer designer—Epidesigner (http://www.epidesigner.com), we found that *GSTA1* and *CD74* possessed no obvious CpG islands or intensive CpG clusters in their promoter regions. We then investigated the methylation level of each CpG site or cluster in the promoters of the other 13 DEGs verified by qPCR (*ANGPTL4*, *CD9*, *NR4A1*, *EDN2*, *PTX3*, *LIF*, *SLC12A8*, *PEX3*, *CYP17A1*, *SERPINE1*, *DIRAS3*, *BNIP3*, *SPP1*) in 16 GC DNA samples (8 PCOS women and 8 controls, the first cohort). We found no significant differences in the methylation levels of CpG sites or clusters of *ANGPTL4*, *CYP17A1*, *PEX3*, *DIRAS3*, *PTX3*, and *SLC12A8* and amplicon for *SPP1* (primer 1) in the GCs between PCOS and control women (Additional file 4: Figure S1a–h). Although two CpG sites in the promoter of *SERPINE1* were hypomethylated, it is probably meaningless for such a low methylation level of these sites (Additional file 4: Figure S1i). Significant differences were found in the methylation levels of some CpG sites or clusters in the promoter regions of *CD9*, *BNIP3*, *EDN2*, *NR4A1*, *LIF*, and *SPP1* (primer 2) between PCOS and control women. To confirm these differences, we further validated the significance of the results in a second cohort consisting of 30 controls and 39 PCOS women. We found that a cluster of CpG sites in the promoter regions of *CD9*, *NR4A1*, *EDN2*, and *BNIP3* were hypomethylated (Fig. 5a–c, e) and that a single CpG site (CpG_8, -780) in the promoter region of LIF was also hypomethylated (Fig. 5d). However, we did not find significant difference in the methylation level of any CpG site of *SPP1* (primer 2) between PCOS and control women (Fig. 5f).

### The influence of promoter methylation status on gene expression

We analyzed the relationship between the methylation levels of CpG sites and gene expression levels of the differentially methylated genes. The expression level of *CD9* was negatively correlated with the average methylation level of *CD9* promoter (*R* = − 0.420, *P* = 0.023) (Fig. 5a). The expression level of *BNIP3* was also, to some extent, correlated with the average promoter methylation status (*R* = − 0.186, *P* = 0.292). However, it is noteworthy that there was a remarkably negative correlation between the expression level and promoter methylation level of *BNIP3* in the control group (*R* = − 0.740, *P* = 0.001), but this correlation was not found in the PCOS group (*R* = 0.23, *P* = 0.923). Therefore, the correlations of these two groups are fully independent of each other. We did not observe significant correlation between the expression level and promoter methylation level of *EDN2*. The expression of *LIF* was negatively correlated with the promoter methylation level (*R* = − 0.565, *P* = 0.001) (Fig. 5d). There is a mildly negative correlation between the expression level and promoter methylation level of *NR4A1* (*R* = − 0.355, *P* = 0.054).

## Discussion

In the present study, we identified 92 DEGs in GCs of PCOS women compared to those of control women. Bioinformatic analysis showed that lipid metabolism was significantly activated in GCs from PCOS women. We also found that global methylation level of DNA in GCs from PCOS women was lower than that of control women. Consistent with these findings, further analysis demonstrated that the differentially methylated genes involved in lipid metabolism and steroid synthesis were hypomethylated and there were negative correlations between the gene expression levels and the promoter methylation levels.

Most women (60–80%) with PCOS have hyperandrogenism [1] and excessive circulating androgens that are mostly from the ovary. Therefore, some physician scientists surmise that abnormal steroidogenesis might be the primary pathogenesis of PCOS [16]; however, such a mechanism needs to be further investigated. GCs around the oocyte are closely associated with the development of the female gamete in the ovary [17] and are involved in steroid synthesis and metabolism [18]. Therefore, profiling the gene expression pattern of GCs and performing deep analysis on the genes related to lipid and steroid metabolism are significant contributions to the field.

GCs from 110 unrelated women with PCOS and 119 normal ovulating women with the comparable age were selected to minimize the influences of age on epigenetic modifications [19]. Cases enrolled in this study showed the main characteristics of PCOS: hyperandrogenism, ovulatory dysfunction, and increased number of antral follicles.

Global profiling of gene expression in target organs with RNA-seq is continuously leading to the establishment of key molecules that potentially participate in the pathophysiology of various diseases. However, due to the high cost, this method is not frequently used as microarrays currently. In a previous study, Kaur et al. employed microarray analysis to identify DEGs in GCs from PCOS and indicated that the DEGs were involved in diabetes, inflammation, cardiovascular diseases, and infertility [20], but few DEGs were related to the major functions of GCs. Crucial for folliculogenesis is GC-oocyte signaling, which relies on free fatty acid beta-oxidation as an energy source for meiosis [21–24]. In our study, 92 DEGs were identified in GCs from PCOS women, and functional analysis showed that 18 DEGs, approximately 20% of all identified DEGs, participated in lipid metabolism. The synthesis of lipid and steroid was significantly activated in the GCs of PCOS women. Increased lipid synthesis in GCs provided steroidal precursor for androgen [25]. Hyperandrogenism, the most constant and prominent diagnostic component of PCOS, is correlated with excess lipid synthesis. The mechanism and causal relationship need to be further explored.

BNIP3 plays a critical function in lipid metabolism [26, 27], and BNIP3 deficiency participates in excess lipid accumulation [27]. The decreased expression of BNIP3 in GCs from PCOS women probably results in excessive synthesis of lipid, which provides precursors for the biosynthesis of androgen in follicles, thus participating in hyperandrogenism. BNIP3 is localized to the outer mitochondrial membrane, where it functions in mitophagy and mitochondrial dynamics. BNIP3, binding directly to LC3 and recruiting the growing autophagosome to mitochondria, is also implicated in the induction of autophagy [28], which often leads to cell death and necrosis. The aberrant low expression of BNIP3 in GCs of PCOS women may be involved in apoptotic deficiency of follicular cells from PCOS women [29], which is associated with polycystic ovarian morphology.

NR4A1, a member of orphan nuclear receptors, initiates gene transcription by various stimuli [30]. It has been described to play a role in lipolysis [31] and promote the transcription of key steroidogenic enzymes in ovarian theca cells, thus enhancing the capacity of the ovarian theca cells to produce androgen [29]. Elevated expression of NR4A1 in GCs may also promote the androgen biosynthesis in PCOS women. Furthermore, NR4A1 is also a clock-related gene, which shows a circadian rhythm in different tissues [32, 33]. Disturbed circadian rhythm could be another point-cut for exploring the pathophysiology of PCOS.

PCOS is considered as a complicated disorder and has certain genetic basis [2]. The heritability of this disorder goes beyond simple Mendelian genetics. Epigenetic and environmental factors have been proposed as confounding factors that modulate the phenotype of genetic basis of PCOS [7, 34]. DNA methylation patterns are neither fixed nor consistent among different tissues and have highly spatio-temporal dynamics that require continuous regulation [35–37].

An approximate 25% reduction was observed in the global DNA methylation level of GCs from PCOS compared with matched controls. This hypomethylation of the genome is believed to result in chromosomal and genomic instability [38–40]. Consistent with reduced global methylation levels in GCs from PCOS women, most of the differentially methylated sites of the genes involved in lipid metabolism and steroid synthesis were also hypomethylated. This is the first epigenetic study to investigate whether global DNA methylation is altered in follicular cells in PCOS women compared with matched controls. Although Xu et al. got a negative result about the global DNA methylation status of peripheral blood between the PCOS and control women [41], it was of great significance to evaluate the global DNA methylation level of follicular cells, especially to quantify the methylation level of a specific CpG site. Recently, Wang et al. carried out a genome-wide DNA methylation analysis in PCOS ovaries and identified 7982 differentially methylated CpG sites [14]. Among those sites, 59.8% were hypermethylated and 40.2% were hypomethylated. This result was neither consistent with nor contradictory to our results. The difference between this study and our own could be interpreted in two aspects. Firstly, the collected tissue by Wang et al. was the ovary tissue, not GCs. Secondly, the control ovaries were obtained from cervical cancer patients who were much older (37.00 ± 5.20 years) than the case group (23.33 ± 2.89 years). A major strength of our study was the comparability of the cases and controls, as age has been reported to affect the methylation status [42]. We matched the two groups and tried to exclude the age-associated epigenetic drift that might affect the global DNA methylation level and the CpG site-specific methylation level of target genes.

The causal relationship between the changes in promoter DNA methylation level and differences in gene expression has been well established [43]. In our study, the expression of the DEGs was at least partially negatively correlated with the average methylation level of the CpG sites in the promoter regions of the differentially methylated genes. DNA methylation regulates gene expression via recruitment of transcriptional factors. However, the correlation was compromised in PCOS women, especially for BNIP3. As for LIF, there was only one differentially methylated CpG site; however, the prediction of transcription factors by TFSEARCH (http://diyhpl.us/~bryan/irc/protocol-online/protocol-cache/TFSEARCH.html) and DBD: Transcription factor prediction database (http://www.transcriptionfactor.org/index.cgi) revealed that the differentially methylated CpG site was the binding site for nuclear respiratory factor 2 (NRF-2). NRF-2, as a transcription factor, is involved in the activation of cytochrome oxidase expression and the nuclear control of mitochondrial function [44, 45]. Loss of methylation in this site may result in abnormal recruitment of NRF-2, which would contribute to aberrant gene expression of LIF.

Folate provides methyl groups for the formation of *S*-adenosylmethionine (SAM) [46], the major methyl donor required for DNA methylation [47]. It is well established that folate supplementation can increase global DNA methylation and affect gene expression [48, 49]. Multiple studies have identified increased homocysteine, an indicator of folate deficiency, in women with PCOS [50, 51], and supplementation with folate is useful to increase the beneficial effect of metformin on the vascular endothelium [52]. Considering the role of global DNA hypomethylation in the GCs of PCOS in our study, we speculate that it is advisable to utilize folate to prevent the global loss of methylation for PCOS women, especially for those undergoing IVF procedure. However, prospective study on this subject should be carried out to determine the appropriate dose and administration duration of folate supplementation.

The limitations of this study should be noted. First, the cells collected in this study are derived from IVF procedure and are a mixture of cumulus and mural GCs and macrophages, as well as are predominantly luteinized mural GCs. It is important to further confirm our findings in non-luteinized GCs. Second, the cohort analyzed by RNA-seq is small and should be considered as a pilot study. Finally, though the enrolled subjects in the two groups were of comparable fasting glucose, the PCOS patients had greater BMI and higher fasting insulin levels and HOMA-IR, which indicates obesity and insulin resistance is more prevalent in the enrolled PCOS women. Altered DNA methylation patterns could be a result of PCOS itself or endocrine and metabolic disturbances of the condition [53, 54]. Therefore, it is of significance to analyze the differential methylation status between women with comparable nutrient status or confirm the epigenetic changes after balancing the endocrine or metabolic profiles of PCOS subjects.

## Conclusions

In summary, this work represents the first study exploring the DNA methylation alterations in GCs of women with PCOS. Genome-wide or gene-specific DNA methylation aberrations identified in our study may be an inducer of changes in gene expression, thus further disrupting the ovarian microenvironment.

## Methods

### Patient selection and sample collection

The datasets during and/or analyzed during the current study are available from the corresponding author on reasonable request. The methods in this work were carried out in accordance with the approved guidelines. One hundred ten PCOS patients, diagnosed according to the Rotterdam Consensus (European Society for Human Reproduction and Embryology/American Society for Reproductive Medicine criteria) [55], and 119 infertile women with tubal blockage (serving as controls) seeking in vitro fertilization (IVF) treatment at the Women’s Hospital of the School of Medicine of Zhejiang University were recruited. The control women met the following inclusion criteria: (1) age between 22 and 36; (2) both ovaries present, without morphological abnormalities; (3) normal ovarian response; (4) menstrual cycle length range between 26 and 33 days; (5) no structural abnormalities of the uterus and ovaries were found by vaginal ultrasound and/or laparoscopy; (6) no diseases affecting gonadotropin and sex steroid secretion, clearance, or excretion; (7) no signs of hyperandrogenism; and (8) no polycystic ovary morphology. And we excluded anyone whose BMI was greater than 25 from our study. The long agonist protocol for controlled ovarian hyperstimulation (COH) and the collection of FFs and GCs, obtained by follicular aspiration from women undergoing oocyte retrieval for IVF, were performed as previously described [7]. The average follicular diameter used for the analysis of FF is 16 mm, and the minimum follicular diameter used to obtain FF is no less than 14 mm.

### Measurement of hormones

Serum fasting glucose levels were measured by glucose oxidase-peroxidase method. The levels of day 3 serum hormones and fasting insulin were measured by chemiluminescence immunoassay (CLIA) in the clinical laboratory of Women’s Hospital, School of Medicine, Zhejiang University. TT levels in serum were measured after exaction [56] using CLIA (Roche, Mannheim, Germany). The hormones in FFs were detected by enzyme-linked immunosorbent assay (ELISA), including androstenedione (Abcam, Cambridge, UK), TT (Abcam, Cambridge, UK), sex hormone-binding globulin (SHBG, R&D Systems, Inc. Minneapolis, MN, USA), and insulin (R&D Systems, Inc. Minneapolis, MN, USA). HOMA-IR was calculated using the formula HOMA-IR = [fasting insulin (mIU/L) × fasting glucose (mmol/L)]/22.5 [57]. Free androgen index (FAI) was calculated as the ratio of TT (in nmol/L)/SHBG (in nmol/L) multiplied by 100 [58]. The values in FFs were from the individual follicle from each woman.

### Transcriptome library preparation and RNA-seq

Total RNA was extracted using the RNeasy® Mini kit according to the manufacturer’s protocol (Qiagen, Hilden, Germany). Total RNA was used to generate the complementary DNA (cDNA) libraries for paired-end sequencing using mRNA-seq Sample Preparation Kit (Illumina). Briefly, 4 μg of total RNA from each sample was used for polyA mRNA selection using polyT oligo-conjugated magnetic beads by two rounds of purification. The cleaved mRNA fragments were reverse transcribed and then converted into double-strand cDNA. Following end repair and A tailing, adapters complementary to sequencing primers were ligated to the ends of DNA fragment. Finally, the ligation products were further purified on 2% agarose gels and 200–250 basepair (bp) fragments were selected for downstream enrichment by 15 cycles of PCR followed by purification using QIAquick PCR purification kit (Qiagen). The libraries were sequenced and quantified with an Illumina HiSeq™ 2000 system.

### Mapping of RNA-seq reads using TopHat

After applying quality control check on the raw sequenced reads using the Fast-QC tool (http://www.bioinformatics.babraham.ac.uk/projects/fastqc/) and removing the adaptor sequences, the clean reads were mapped and aligned to the human genome (hg19) using TopHat program (v2.0.6) [59]. TopHat allows multiple alignments per read (up to 20 by default) and a maximum of two mismatches when mapping the reads to the reference. TopHat builds a database of potential splice junctions and confirms these by comparing the previously unmapped reads against the database of putative junctions. The default parameters for the TopHat method were used.

### DEG dataset and functional analyses

All genes and transcripts have been assigned a relative coverage rate as measured by fragments per kilobase per million (FPKM). To define genes up- or downregulated between the two groups, the log2 of the proportion between the sum of the FPKM for all gene transcripts and the same sum in control condition was taken as a measure of change in gene expression. The *P* value was obtained by performing a Fisher exact test (number of reads mapped to the gene and number of reads mapped to all other genes in GCs from PCOS women versus the controls), and the FDR was calculated to correct the *P* value. The smaller FDR indicates the smaller error in judging the *P* value. A significant DEG dataset was determined with a fold change (FC) cutoff of 1.5, *P* < 0.05, and FDR < 0.05.

IPA software (Qiagen, Redwood 185 City, CA) was used to identify the top biological functions associated with the DEG dataset, using Fisher’s exact test to determine the probability that each biological function assigned to the DEGs is explained by chance alone. Downstream effect analysis was used to predict downstream biological processes and infer their activation state based on the observed gene expression changes in DEG dataset. A *z* score was calculated and used to infer the activation states (“increased” or “decreased”) of implicated biological processes [60].

### Reverse transcription and quantitative real-time PCR (qPCR)

Expression level of 15 genes was measured by qPCR in GCs. Total RNA was isolated using the RNAiso™ Reagent, and cDNA was prepared using the PrimeScript™ RT reagent kit with gDNA Eraser. qPCR analysis was carried out to determine the mRNA expression of the related genes using the SYBR® Premix Ex TaqTM (Tli RNaseH Plus) system (TAKARA, Dalian, China) in an Applied Biosystems ViiA™ 7 Real-Time PCR System (ABI, Carlsbad, CA). The amplification thermal cycling conditions were 95 °C for 10 s for one cycle, 95 °C for 5 s, and 60 °C for 30 s, followed by 40 cycles. After PCR, a dissociation curve was constructed at 95 °C for 15 s, 60 °C for 15 s, and 95 °C for 15 s for detection of PCR product specificity. The glyceraldehyde-3-phosphate dehydrogenase (GAPDH) was served as the internal control. Optimal qPCR assay for the gene was amplified using the corresponding primers (listed in Additional file 1: Table S1).

### DNA preparation

Genomic DNA from GCs of PCOS patients and controls was isolated using the QIAamp DNA Mini Kit (Qiagen, Hilden, Germany) as recommended by the manufacturer.

### Quantification of 5mC

Global methylation of GCs was determined by 5-methylcytosine (5-mC) DNA ELISA kit (Zymo Research, Irvine, CA). One hundred nanogram genomic DNA from GCs was subjected to the quantification of 5-mC, following the manufacturer’s instructions. All the samples (or repeats) were loaded with the same amount of DNA in the assay plate. This was followed by the addition of primary (monoclonal antibody specific to 5-mC) and secondary antibody. The absorbance of each sample was read at 405 nm and converted into percentage of 5-mC, according to a standard curve generated by serial dilutions of the 5-mC DNA positive and negative control provided with the kit. The results were taken as average of two independent experiments. This analysis provides the levels of global DNA methylation and not specific to any particular gene.

### CT conversion and MassArray EpiTYPER quantitative DNA methylation analysis

Bisulfite conversion of the genomic DNA was performed with the EZ DNA CT Conversion Reagent, Zymo Research Corporation (Irvine, CA, USA) according to the manufacturer’s protocol. Quantitative DNA methylation analysis was performed with MassArray EpiTyper (Sequenom, San Diego, CA, USA) as described previously [61]. The promoter regions of the target genes were analyzed. Amplicons cover the regions of the corresponding CpG sites, and the target regions are shown in the corresponding figures. Primers were designed using the online software Epidesigner (http://www.epidesigner.com) shown in Additional file 1: Table S2. The quantitative methylation data for each CpG site or aggregates of multiple CpG sites obtained from MassArray were analyzed on the EpiTYPER software (Sequenom).

### Statistical analysis

Statistical analysis is performed using the software SPSS version 19.0 software (SPSS Inc., Chicago, IL, USA). The comparisons between two groups were performed with Student’s *t* test or non-parametric Mann-Whitney *U* tests. The correlations between mRNA expression levels and CpG site methylation levels of a specific gene were analyzed using Pearson’s correlation. *P* < 0.05 was considered statistically significant.

## Additional files

Additional file 1: The primer sets used in this study and clinic characteristics of patients used for Transcriptome Sequencing. (DOCX 30 kb)

Additional file 2: Dataset S1. All genes detected by Transcriptome Sequencing. (XLS 4329 kb)

Additional file 3: Dataset S2. 92 genes significantly differentially expressed in GCs of PCOS compared with controls. (XLS 68 kb)

Additional file 4: Figure S1. MassARRAY Quantitative methylation analysis of the CpG sites in the promoter of target genes. Values are mean ± SE. Eight GC genomic DNA from controls and 8 GC genomic DNA from PCOS women were analyzed. *P* value was determined by Mann-Whitney *U* test. *P < 0.05. (EPS 2291 kb)

## Abbreviations

5-mC: 5-Methylcytosine
AFC: Antral follicle count
BMI: Body mass index
CLIA: Chemiluminescence immunoassay
COH: Controlled ovarian hyperstimulation
DEGs: Differentially expressed genes
FAI: Free androgen index
FDR: False discovery rate
FF: Follicular fluid
FPKM: Fragments per kilobase per million
FSH: Follicle-stimulating hormone
GAPHD: Glyceraldehyde-3-phosphate dehydrogenase
GCs: Granulosa cells
HOMA-IR: Homeostasis model assessment of insulin resistance
IPA: Ingenuity Pathway Analysis
IVF: In vitro fertilization
LH: Luteinizing hormone
PCOS: Polycystic ovary syndrome
qPCR: Quantitative polymerase chain reaction
SAM: *S*-Adenosylmethionine
SHBG: Sex hormone-binding globulin
TT: Total testosterone
