# Supplementary material for: Aberrant expression and DNA methylation of lipid metabolism genes in PCOS: a new insight into its pathogenesis
Source: Clin Epigenetics. 2018 Jan 12;10:6. doi: 10.1186/s13148-018-0442-y (PMC5767000; doi:10.1186/s13148-018-0442-y)
Supplement: Supplementary file 1 — The primer sets used in this study and clinic characteristics of patients used for Transcriptome Sequencing. (DOCX 30 kb) [file 13148_2018_442_MOESM1_ESM.docx]

**Additional file 1: Table S1.** The primer sets of real-time PCR assay used in this study.

| **Gene Name** | **Primer Name** | **Sequence (5′ –3′)** |
| --- | --- | --- |
| SLC12A8 | SLC12A8-F | CTGGTGTCCTTCGTCATCCTG |
|  | SLC12A8-R | CACCTGCAACACACTGTCCA |
| NR4A1 | NR4A1-F | ATGCCCTGTATCCAAGCCC |
|  | NR4A1-R | GTGTAGCCGTCCATGAAGGT |
| PEX3 | PEX3-F | CCAAGCACGACGACAATATCA |
|  | PEX3-R | TCAGTGTTGGAAGCATGGACA |
| LIF | LIF-F | CCAACGTGACGGACTTCCC |
|  | LIF-R | TACACGACTATGCGGTACAGC |
| DIRAS3 | DIRAS3-F | GAGTGAGCTCTTGGGGTGTC |
|  | DIRAS3-R | TTGGGATTCGGAGGGGAGAT |
| ANGPTL4 | ANGPTL4-F | GGCTCAGTGGACTTCAACCG |
|  | ANGPTL4-R | CCGTGATGCTATGCACCTTCT |
| SPP1 | SPP1-F | GGAGTTGAATGGTGCATACAAGG |
|  | SPP1-R | CCACGGCTGTCCCAATCAG |
| SERPINE1 | SERPINE1-F | ACCGCAACGTGGTTTTCTCA |
|  | SERPINE1-R | TTGAATCCCATAGCTGCTTGAAT |
| GSTA1 | GSTA1-F | CTGCCCGTATGTCCACCTG |
|  | GSTA1-R | AGCTCCTCGACGTAGTAGAGA |
| BNIP3 | BNIP3-F | CAGGGCTCCTGGGTAGAACT |
|  | BNIP3-R | CTACTCCGTCCAGACTCATGC |
| CD74 | CD74-F | GACGAGAACGGCAACTATCTG |
|  | CD74-R | GTTGGGGAAGACACACCAGC |
| CD9 | CD9-F | TTCCTCTTGGTGATATTCGCCA |
|  | CD9-R | AGTTCAACGCATAGTGGATGG |
| DHRS9 | DHRS9-F | CTGTGGACTCGTAAAGGAAAACT |
|  | DHRS9-R | GCAGCGATTACATGAAATCCCT |
| EDN2 | EDN2-F | CGTCCTCATCTCATGCCCAAG |
|  | EDN2-R | AGGCCGTAAGGAGCTGTCT |
| GAPDH | GAPDH-F | ACAACTTTGGTATCGTGGAAGG |
|  | GAPDH-R | GCCATCACGCCACAGTTTC |
| PTX3 | PTX3-F | AGGCTTGAGTCTTTTAGTGCC |
|  | PTX3-R | ATGGATTCCTCTTTGTGCCATAG |
| CYP17A1 | CYP17A1-F | TATGGCCCCATCTATTCGGTT |
|  | CYP17A1-R | GCGATACCCTTACGGTTGTTG |

**Additional file 1: Table S2.** The primer sets of methylation analysis used in this study.

| **Gene Name** | **Region** | **Primer Name** | **Sequence (5′ –3′)** |
| --- | --- | --- | --- |
| ANGPTL4 | chr19:8428858-8429211 | ANGPTL4_F | aggaagagagGTTTATTTTTTTAGGTAGTTTGGGAG |
|  |  | ANGPTL4_R | cagtaatacgactcactatagggagaaggctAAAAAACCACAATTTAACATCCCC |
| BNIP3 | chr10:133794952-133795287 | BNIP3_F | aggaagagagGATGTAGGAGGAGAGTTTGTAGGGT |
|  |  | BNIP3_R | cagtaatacgactcactatagggagaaggctTTCACCTCCAAATAAAATTCTAACTC |
| CD9 | chr12:6309644-6310070 | CD9_F | aggaagagagGAGGGATTTGAGTTGGGAGTAGATA |
|  |  | CD9_R | cagtaatacgactcactatagggagaaggctCCAAACTAAATTAACCCTCACCATA |
| CYP17A1 | chr10:104597634-104598030 | CYP17A1_F | aggaagagagGGTTTGAGGTTTAGGTGGGTATTAT |
|  |  | CYP17A1_R | cagtaatacgactcactatagggagaaggctACCCTCCAACCCATATAATTTTAAC |
| DIRAS3 | chr1:68512617-68512891 | DIRAS3_F | aggaagagagTTTTTTTGGTGATTGAGTAGATTAGG |
|  |  | DIRAS3_R | cagtaatacgactcactatagggagaaggctACCCCACAAAAAAATCAAAAATTAC |
| EDN2 | chr1:41950191-41950550 | EDN2_F | aggaagagagGGTGTTTTAGGTATAGAGGGAGGAA |
|  |  | EDN2_R | cagtaatacgactcactatagggagaaggctAAAACCAAAACAACTCACCTTCATA |
| LIF | chr22:30643339-30643656 | LIF_F | aggaagagagTATTGTTATTGGGTTTTAAGGGGTT |
|  |  | LIF_R | cagtaatacgactcactatagggagaaggctAAAAAAACCAACCACTTCTCAATTT |
| NR4A1 | chr12:52444910-52445274 | NR4A1_F | aggaagagagTAGTGGGTTTGGGAGTTGTTATTTT |
|  |  | NR4A1_R | cagtaatacgactcactatagggagaaggctAATTCTTCTATACACTCCCCCAAAT |
| PEX3 | chr6:143771647-143772108 | PEX3_F | aggaagagagTGAGGGTTGGAGAAAGGTTTTAT |
|  |  | PEX3_R | cagtaatacgactcactatagggagaaggctCTAACCCAAATAACCCAACAAAAA |
| PTX3 | chr3:157155500-6310070 | PTX3_F | aggaagagagAGGTTAGGTTGATTAGTGTTTTGGA |
|  |  | PTX3_R | cagtaatacgactcactatagggagaaggctCCCATAAAAACTTCCCCTAAAAAAT |
| SERPINE1 | chr7:100769676-100770164 | SERPINE1_F | aggaagagagTTTTAGGGGTATAGAGAGAGTTTGGA |
|  |  | SERPINE1_R | cagtaatacgactcactatagggagaaggctACAAACCCCAATAACCTTAACCTAA |
| SLC12A8 | chr3:124931830-124932116 | SLC12A8_F | aggaagagagGTTTTTAGAGTTTGGGGTTATTTGA |
|  |  | SLC12A8_R | cagtaatacgactcactatagggagaaggctAAATAACCAACCACTACCTCTCCC |
| SPP1_1 | chr4:88894807-88895002 | SPP1_1F | aggaagagagTGGAGAGTTTTATAGTTGTATATAGTTTTG |
|  |  | SPP1_1R | cagtaatacgactcactatagggagaaggctACCCAACCATCTTAAAATAAACCAT |
| SPP1_2 | chr4:88896593-88896997 | SPP1_2F | aggaagagagGGGATTTTTAAGTGTTTTTTTTGGA |
|  |  | SPP1_2R | cagtaatacgactcactatagggagaaggctAAAATTCAACTAAATACACAACCCAA |
|  |  |  |  |

**Additional file 1: Table S3.** Demographic data and clinic characteristics of the IVF patients used for Transcriptome Sequencing

| Items | C1 | C2 | C3 |  | P1 | P2 | P3 | *P* value |
| --- | --- | --- | --- | --- | --- | --- | --- | --- |
| Age | 33 | 25 | 27 |  | 29 | 32 | 32 | 0.364 |
| Body Mass Index | 21.36 | 20.7 | 20.03 |  | 20.83 | 22.65 | 22.38 | 0.141 |
| Cycle length | 28 | 28 | 30 |  | 60 | 50 | 50 | 0.002 |
| Day3 LH/FSH | 0.66 | 0.81 | 0.79 |  | 3.66 | 2.11 | 0.7 | 0.178 |
| Day3 TT (nmol/L) | 0.9 | 0.4 | 0.9 |  | 3.68 | 2.3 | 3.45 | 0.006 |
| Day3 DHEA-S (umol/L) | 4.5 | 4.4 | 9.3 |  | 8.9 | 8.8 | 11.7 | 0.117 |
| Antral follicle count | 7 | 14 | 10 |  | 18 | 24 | 24 | 0.015 |
| Androstenedione (ng/ml) in FF | 5.45 | 3.37 | 6.94 |  | 35.35 | 66.96 | 13.71 | 0.097 |
| TT (ng/ml) in FF | 5.26 | 3.2 | 5.76 |  | 21.95 | 35.91 | 16.8 | 0.025 |
| SHBG (nmol/L) in FF | 75.11 | 57.74 | 59.58 |  | 58.91 | 42.52 | 40.51 | 0.104 |
| FAI in FF | 24.28 | 19.16 | 33.5 |  | 129.17 | 292.77 | 143.79 | 0.036 |

Note: Day 3, the third day of spontaneous menstrual cycle; LH, luteinizing hormone; FSH, follicle stimulating hormone; TT, total testosterone; DHEA-S, dehydroepiandrosterone-sulfate; FF, follicular fluid; SHBG, sex hormone binding globulin; FAI, free androgen index; *P* value was determined by Student *t* tests.
